# Applications of CRISPR-Cas9 Technology to Genome Editing in Glioblastoma Multiforme

**DOI:** 10.3390/cells10092342

**Published:** 2021-09-07

**Authors:** Nadia Al-Sammarraie, Swapan K. Ray

**Affiliations:** Department of Pathology, Microbiology and Immunology, School of Medicine, University of South Carolina, Columbia, SC 29209, USA; nadia.al-sammarraie@uscmed.sc.edu

**Keywords:** glioblastoma multiforme (GBM), CRISPR-Cas9 genome editing, apoptosis, proliferation, autophagy, angiogenesis, cell invasion and migration

## Abstract

Glioblastoma multiforme (GBM) is an aggressive malignancy of the brain and spinal cord with a poor life expectancy. The low survivability of GBM patients can be attributed, in part, to its heterogeneity and the presence of multiple genetic alterations causing rapid tumor growth and resistance to conventional therapy. The Clustered Regularly Interspaced Short Palindromic Repeats (CRISPR)-CRISPR associated (Cas) nuclease 9 (CRISPR-Cas9) system is a cost-effective and reliable gene editing technology, which is widely used in cancer research. It leads to novel discoveries of various oncogenes that regulate autophagy, angiogenesis, and invasion and play important role in pathogenesis of various malignancies, including GBM. In this review article, we first describe the principle and methods of delivery of CRISPR-Cas9 genome editing. Second, we summarize the current knowledge and major applications of CRISPR-Cas9 to identifying and modifying the genetic regulators of the hallmark of GBM. Lastly, we elucidate the major limitations of current CRISPR-Cas9 technology in the GBM field and the future perspectives. CRISPR-Cas9 genome editing aids in identifying novel coding and non-coding transcriptional regulators of the hallmarks of GBM particularly in vitro, while work using in vivo systems requires further investigation.

## 1. Introduction

Glioblastoma multiforme (GBM) is an aggressive primary tumor, which arises from the abnormal astroglial cells in the brain in most cases as well as in the spinal cord in less often cases [1,2]. The incidence of GBM is approximately less than 10 per 100,000 individuals worldwide, and a significant number of the GBM patients show a low survival rate of 14 months or less [3,4]. The heterogenous nature of the GBM contributes to its therapy-resistance and poor prognosis; hence, identifying genetic regulations of the hallmarks of this malignant disease may help device effective treatments [5,6].

Almost all GBM patients are presented with the high-grade of the disease, which is characterized by rapid recurrence after surgery and resistance to radiotherapy and chemotherapy [7,8]. Resistance of GBM to therapies is a major challenge for treatment of the patients and it can result from several mechanisms, which collectively constitute the hallmarks of this malignancy [7,8]. The resistance mechanisms can either accelerate initial tumor growth or potentiate regrowth of the more resistant tumor after the treatment [7,8]. One of the major hallmarks of GBM is the presence of GBM stem cells (GSCs), which are genetically heterogeneous cells with more distinct properties than primary tumor cells, and they play critical roles in disease recurrence and therapy resistance [9,10]. Sustained proliferative signals is another major cause of resistance, which results from aberrant expression of the growth and trophic factors [11]. Escaping the programed cell death or apoptosis and activating the pro-survival pathways are other major mechanisms of therapy resistance in GBM [12]. Autophagy or recycling of cellular building blocks is also an important survival mechanism that is activated in GBM and it is one of the major causes of therapy resistance [13]. Aberrant inflammation and immune response are correlated with rapid progression and resistance to therapy [14,15]. Angiogenesis or aberrant new blood vessel formation is also correlated with rapid progression and therapy resistance in GBM [15,16]. Finally, interaction between tumor cells and the surrounding microenvironment potentiates the migratory and invasive properties of the tumor cells, contributing to rapid relapse of GBM and poor outcome [17,18,19].

Genetic knockout, knock in, and overexpression have been widely used to screen for the molecular pathways that govern the hallmarks of GBM and to study the function of various genes in pathogenesis and progression of this malignant disease [20,21,22,23]. In the past, scientists used ‘gene targeting’ for changing the genome in the specific places with addition or deletion of either entire genes or single bases. Although gene targeting has highly been useful in understanding the function of specific genes, this technology takes long time to make a mutant gene and it is expensive. Subsequently, several ‘gene editing’ technologies such as Transcription Activator-Like Effector Nucleases (TALENs) and Zinc-Finger Nucleases (ZFNs) have been discovered to improve the gene targeting to a great extent. Still scientists were looking for a cheaper and quicker gene editing technology than TALENs and ZNFs. The Clustered Regularly Interspaced Short Palindromic Repeat (CRISPR)-CRISPR associated (Cas) nuclease 9 (CRISPR-Cas9) system is the latest gene editing technology [20], which stands out as the fastest, cheapest, highly versatile, and most reliable gene editing tool for using widely to discover genetic alterations, oncogenic targets, and epigenetic regulation. Currently, CRISPR-Cas9 system is the number one choice for editing genes or genome in various cancers including GBM [21,22,23,24].

**Figure 1:**
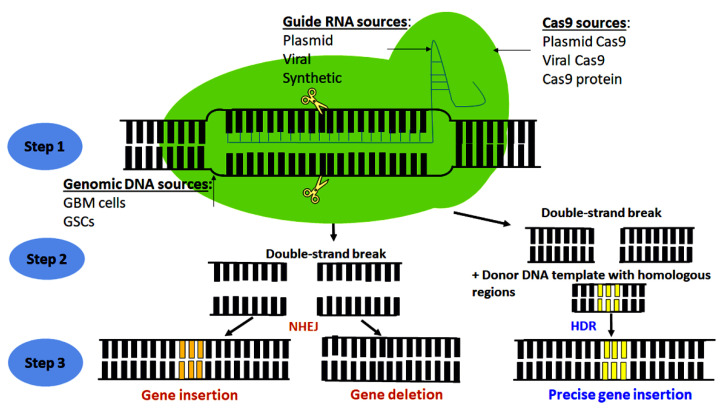
Principle of CRISPR-Cas9 genome editing and sources of its major elements for application to GBM research. CRISPR-Cas9 genome editing involves three major steps: Step 1, guide RNA binds to complementary sequence in the gene of interest; Step 2, Cas9 performs double-strand DNA break; Step 3, activation of non-homologous end joining (NHEJ) mechanism repairs DNA by directly ligating double-strand DNA break ends after DNA sequences are inserted or deleted, or activation of homology-directed repair (HDR) mechanism inserts the targeted DNA sequence in the presence of donor DNA template with homology regions to the cut-ends resulting in more precise gene insertion. Majority of CRISPR-Cas9 genome editing applications were performed in GBM cell lines or GSCs. The sources of guide RNA used in GBM research were variable such as plasmid, viral, and synthetic. Similarly, the sources of Cas9 were varying including plasmid Cas9, viral Cas9, and Cas9 protein.

**Figure 2:**
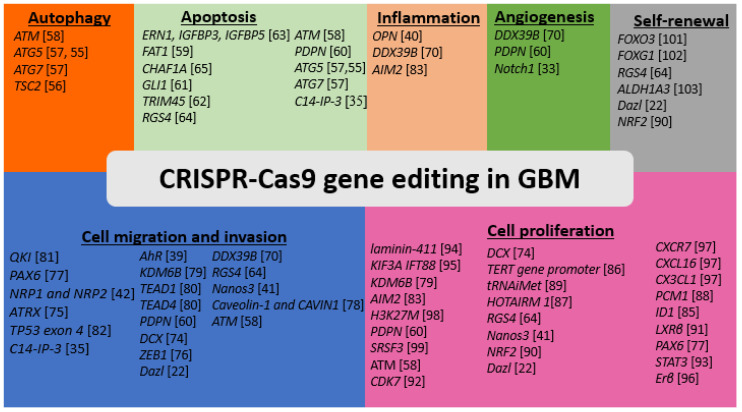
Application of CRISPR-Cas9 genome editing to identifying genes that correlate with different hallmarks of GBM. CRISPR-Cas9 mediated gene knockdown, knockout, and overexpression have been used for identifying several genes that have unique and overlapping roles in GBM development, progression, and recurrence. The listed genes with their references are the ones already edited using CRISPR-Cas9 genome editing technology in GBM research.

In this article, we will first briefly discuss the basic principle of CRISPR-Cas9 system (Figure 1) and its application to GBM in revealing the function of genes that contribute to maintaining tumor cell growth and stemness, escaping the programmed cell death or apoptosis, inducing autophagy, promoting angiogenesis, deregulating immune response and inflammation, and potentiating cell invasion and metastasis (Figure 2). Finally, we will discuss the pitfalls and current limitations of CRISPR-Cas9 gene editing systems in GBM research and future prospective.

## 2. Principle of CRISPR-Cas9 Genome Editing Technology

CRISPR-Cas9 is a naturally occurring protective immune system, which is found as the repeated DNA clusters of 21–47 bp in bacteria and Archaea [25,26,27,28]. In these prokaryotes, CRISPR-Cas9 provides an internal defense mechanism by recognizing and eliminating foreign viral DNA [25,26,27,28]. When the virus attacks the prokaryote for the first time, it introduces its DNA that triggers the immune system of the prokaryote to generate small fragments of DNA called CRISPR arrays [25,26,27,28]. The CRISPR arrays help the bacteria recognize subsequent viral invasion and transcribe guide RNA targeting the viral DNA, which is then degraded by an endonuclease enzyme called the Cas9 protein [25,26,27,28]. Using similar approach, CRISPR-Cas 9 gene editing technology has been developed and widely used to study the functions of various genes that are responsible for GBM development and progression both in vitro and in vivo.

CRISPR-Cas9 gene editing technology is composed of two main elements: the guide RNA and the Cas9 endonuclease enzyme [29,30]. The guide RNA is a synthetic complex made by hybridization of two different RNAs: the CRISPR RNA (crRNA), which has a complementary nucleotide sequence to the target DNA; and the trans-activating CRISPR RNA (tracrRNA), which binds and activates the Cas9 nuclease [31,32]. On one side, the guide RNA binds to a complimentary sequence in the DNA; and on other side, it binds and directs Cas9 endonuclease enzyme to the target DNA segment to perform the genome editing [30,31,32]. In addition to the regular Cas9 nuclease that results in double-strand DNA break, Cas9 nickase has been developed via mutagenesis of the regular Cas9 nuclease, and Cas9 nickase enables genome editing via single-strand DNA break that permits more precise genome editing and minimizes the off-target effects of the Cas9 nuclease [31].

The Cas9 enzymes used in GBM research were obtained from different sources or expression vectors such as Cas9-expressing lentiviral vector, Cas9-expressing plasmid vector, or Cas9 synthetic protein [22,23,33]. Similarly, the guide RNA molecules were obtained from multiple sources such as lentiviral, plasmid, or synthetically derived guide single strand RNA [22,23,33]. CRISPR-Cas9 systems were delivered into GBM cells using viral (lentiviral mediated) or non-viral lipid-mediated (Lipofectamine 3000) methods [34,35]. After the delivery into the cells, the guide RNA that contains the targeting RNA sequence becomes complementary to the gene of interest to be edited [30,31,32]. Cas9 endonuclease then create a double-strand DNA break in the targeted region of the genome to edit the gene of interest [30,31,32].

Once the double-strand break is generated, a DNA repair machinery is activated to form either a non-homologous end joining (NHEJ) or a HDR [36,37,38]. NHEJ is ‘non-homologous repair’, in which the DNA break ends are directly ligated without requiring a homologous template, in contrast to HDR that requires a homologous sequence for guiding the DNA repair [36,37,38]. However, NHEJ results is imprecise joining of two ends of DNA, while HDR results in precise insertion due to involvement of a designed DNA template [36,37,38]. Puromycin or fluorescence-activated cell sorting (FACS) is commonly used for selection of the transfected cells [34,39,40] while further validation of gene editing is commonly performed using quantitative polymerase chain reaction (qPCR) or Western blotting [40,41,42].

## 3. Genome-Wide CRISPR-Cas9 Screens in GBM Research

CRISPR-Cas9 screens have been used in vitro and in vivo for identifying the novel biomarkers, oncogenic drivers, mechanisms of chemotherapy resistance, and genes that make tumor cells more responsive to standard or synergistic therapy. CRISPR-Cas9 genome-wide screening used in GBM research includes either knockouts or interference approaches; and they are performed mostly on GBM cell lines, GSCs, and less commonly are applied to cerebral organoid or in vivo in mice. CRISPR guide RNA library used in GBM research are either coding or non-coding and are commonly transfected into GBM models using viral transduction (Table 1).

To identify new prognostic biomarkers and factors that sensitize tumor cells to chemotherapy, a group of investigators used CRISPR-Cas9 mediated genome-wide knockouts to identify ribosomal protein subunits 11, 16, and 18 as important biomarkers in GBM cell lines in response to treatment with topoisomerase II poisons [43]. Also, they identified that loss of ribosomal subunit 11 correlated with resistance to cell death in response to the common chemotherapeutic agents such as etoposide and doxorubicin [43]. Another group used CRISPR-Cas9 mediated screen to identify NF-κB (nuclear factor kappa-light-chain-enhancer of activated B cells) and E2F6 (E2F transcription factor 6) genes as one of the major underlying molecular mechanisms of resistance to temozolomide (TMZ), an orally administered alkylating chemotherapeutic agent, in epidermal growth factor receptor (EGFR) variant III (EGFRvIII)-expressing U87MG cells [44]. Another study used in vivo CRISPR-Cas9 screen to identify GBM suppressor genes in mice [45]. In a different study, use of CRISPR-Cas9 mediated genome-wide knockout screen identified mitogen-activated protein kinase kinase kinase kinase-4 (MAP4K4) as an important regulator of invasion in U138MG cells [46]. Besides, use of an in vivo CRISPR-Cas9 knockout screen in mice identified genetic alterations in surface proteins of CD8+ T cells regulating T cell immunotherapy in GBM [47].

To identify genetic regulation of GBM stemness, an investigation used CRISPR-Cas9 screen to identify key regulators or transcription factors that controlled growth, stemness, and TMZ resistance in GSCs [48]. Employment of CRISPR-Cas9 mediated genome-wide screen identified new molecular regulator of cancer stem cells in three-dimensional bioprinted complex systems, which conferred the interaction between GBM cells and the surrounding microenvironment [49]. Interestingly, another group has used CRISPR-Cas9 knockout screen to identify the loss of redundancy between PKMYT1 (protein kinase, membrane associated tyrosine/threonine 1) and WEE1 (‘wee phenotype’ 1 protein kinase), which are major regulators of mitosis, in GSCs when compared with neural stem cells (NSCs), enhancing growth in GSCs [50].

Apart from transcriptional screen, CRISPR-Cas9 systems have been used to explore the role of non-coding regions in pathogenesis of GBM. For example, use of CRISPR-Cas9 interference screen identified genetic alterations in long non-coding RNAs (lncRNAs) that could control growth in U87MG cells and sensitize them to therapeutic doses of ionizing radiations [51]. Also, use of CRISPR-Cas9 interference screen identified amplification of non-coding region in the DNA that could regulate the co-amplified oncogenes in GBM [52]. All these revolutionized and provided insights into functional correlation among heterogenous GBM mutations, which could be potential therapeutic targets.

**Table 1:** CRISPR-Cas9 genome-wide screens used in GBM.

Tumor Model	Type of Screen	References
SNB19	Genome-scale CRISPR knockout screen	[43]
U138MG	Large-scale CRISPR-Cas9 mediated loss of function screen	[46]
GSCs	Whole-genome CRISPR screening	[49]
U87MG	CRISPR interference (CRISPRi) screen	[51]
GBM3565, GSC23	CRISPRi screen	[52]
Patient-derived GSCs	Genome-wide CRISPR-Cas9 screens	[48]
Mice	In vivo CRISPR screen	[45]
Patient-derived GSCs and human NSCs	Genome-wide CRISPR-Cas9 screen	[50]
U87MG and U87MG-EGFRvIII cells	Pooled genome wide CRISPR screening	[44]

## 4. Application of CRISPR-Cas9 to Identifying Genetic Regulators of Autophagy in GBM

Autophagy is a catabolic mechanism of recycling intracellular components and organelles by normal and tumor cells [53]. Autophagy includes a sequence of events from autophagosome formation to fusion with lysosome and finally lysis of the engulfed materials [53]. In GBM, autophagy plays controversial roles in developing and advancing this disease; however, several studies have correlated autophagy activation in GBM with aggressive disease and therapy resistance [13,54]. CRISPR-Cas9 system has been used in GBM research to identify transcriptional regulation, biological function, and interactions of genes that control autophagy activation and autophagy flux in GBM (Table 2).

A study has shown inhibition of autophagy induction by CRISPR-Cas9-mediated ATG5 gene knockout in TGS01 and TGS04 cells in conjunction with a calcium mobilization agent (nigericin) that works together to increase mitochondrial reactive oxygen species for cell death [55]. Another study showed that CRISPR-Cas9 mediated knockout of TSC2 (Tuberous Sclerosis 2) gene, an autophagy promoting molecule, in GBM LN18 cells rendering them to be more susceptible to cell death in response to photodynamic therapy (PDT) [56]. In contrast, another study reports that ATG5 and ATG7, both of which are autophagy related genes, are important for cell death in GBM while CRISPR-Cas9 mediated knockout of ATG5 and ATG7 in GBM MZ-54 cells protect the cells from cell death when compared with control cells in response to various autophagy inducers (loperamide, pimozide, and STF-62247) [57]. The difference in the results obtained from these studies could be attributed to the heterogenous nature of GBM and the different effect of autophagy regulatory gene knockout in different cell lines used. Also, it could be due to synergetic or antagonistic effect of different combination therapies that were used along with ATG5 knockout, resulting in different effects on GBM cell death. This also could explain why autophagy induction or activation remained controversial in GBM treatment. Similarly, CRISPR-Cas9 mediated knockout of ataxia-telangiectasia mutated (ATM) gene, a tumor suppressor, in two human GBM cell lines such as LN18 and LN229 potentiated autophagy and increased their responsiveness to platinum treatment [58].

**Table 2:** CRISPR-Cas9 mediated knockouts of autophagy genes in GBM.

Target Gene	Type of CRISPR-Cas9 Mediated Genome Editing	GBM Model	References
ATM	Knockout	LN18, LN229	[58]
ATG5 and ATG7	Knockout	MZ-54 GBM cells	[57]
ATG5	Knockout	TGS01 or TGS04	[55]
TSC2	Knockout	LN18 cells	[56]

## 5. Application of CRISPR-Cas9 to Identifying genetic Regulators of Apoptosis in GBM

Escaping the programed cell death or apoptosis plays a pivotal role in therapy resistance in GBM [12]. Escaping apoptosis can result from several mechanisms. For example, loss of tumor suppressor genes, activation of growth signals such as mechanistic target of rapamycin (mTOR) signaling, and upregulation of anti-apoptotic signals [12]. CRISPR-Cas9 systems have been used widely to study transcriptional and epigenetic regulators of cell death in GBM and to identify genetic and molecular mechanisms that induce or enhance apoptosis in the chemo-resistant tumor (Table 3).

CRISPR-Cas9 mediated knockouts have been used to identify the role of membrane proteins, which are highly expressed for initiating apoptosis in therapy resistant GBM. Application of CRISPR-Cas9 mediated knockout of FAT1 (fat atypical cadherin 1) gene in GBM U251 cells made the cells more prone to receptor-mediated apoptosis [59]. Besides, another group performed CRISPR-Cas9 mediated knockout of the glycoprotein podoplanin (PDPN) gene in several GBM cell types including GBMF2, GBMF3, LN308, and LN319 [60]. However, they found no changes in apoptosis in knockouts when compared with intact controls but since it was associated with aggressive tumor, it could be used as a prognostic biomarker [60]. CRISPR-Cas9 mediated knockouts have also been used to identify the genetic and transcriptional regulations of pro-survival pathways and the role of transcription repressor in potentiating apoptosis in GBM cells or GSCs. Use of CRISPR-Cas9 to knockout the tumor suppressor ATM gene in GMB LN18 and LN229 cell lines in conjunction with cisplatin treatment for cell death [58]. Use of CRISPR-Cas9 to knockout GLI1, a Sonic Hedgehog-related transcription factor, in GBM 28 in conjunction with treatment with penflurido, an anti-psychotic agent, potentiated activation of caspase-3 for induction of cell death [61]. Another study used CRISPR-Cas 9 knockout to identify the function of tripartite motif-containing protein 45 (TRIM45) gene in GBM U87MG and LN229 cell lines and found that the knockdown inhibited apoptosis and potentiated the growth of GBM cells [62]. A different group used CRISPR-Cas9 to knockout the Unfolded Protein Response (UPR) genes ERN1, IGFBP3, and IGFBP5 in GBM U251 cells resulting in their increased susceptibility for cell death in response to an endoplasmic reticulum stress-inducing drug (12 ADT) [63]. Besides, another group used CRISPR-Cas9 to knockout and identify the function of the regulator of G-protein signaling 4 (RGS4) gene in cancer stem cells. RGS4 is a negative regulator of G-protein signaling and its knockout induces apoptosis in GSC20 cells [64].

Furthermore, CRISPR-Cas9 have been employed to identify epigenetic regulation of apoptosis in GBM cells. Studies showed that CRISPR-Cas9 mediated activation of Chromosome 14 Internal Promoter 3 (C14-IP-3), internal promotor of C14MC miRNAs, in the human GBM LN229 cell line activated apoptosis [35]. It has been shown that CRISPR-Cas9 mediated knockout of Chromatin assembly factor 1 subunit A (CHAF1A) gene, which is associated with poor prognosis of GBM, can trigger apoptosis in two GBM cell lines U251 and U87MG [65].

All these results are very exciting as they show the success of CRISPR-Cas9 technology in inducing apoptosis in different GBM cell lines. Future studies need to use CRISPR-Cas9 technology for editing genes for promotion and enhancement of induction of apoptosis in GBM in animal models.

**Table 3:** CRISPR-Cas9 mediated gene editing of apoptosis genes in GBM.

Target Gene	Type of CRISPR-Cas9 Mediated Genome Editing	GBM Model	References
ERN1 IGFBP3 IGFBP5	Knockout	U251	[63]
FAT1	Knockout	U251MG	[59]
CHAF1A	Knockout	U251MG, U87MG	[65]
GLI1	Knock down	GBM28	[61]
TRIM45	Knockout	U87MG LN229	[62]
RGS4	Knockout	GSC20	[64]
ATM	Knockouts	LN18, LN229	[58]
Podoplanin (PDPN)	Knockout	GBMF2, GBMF3, LN308, LN319	[60]
ATG5 ATG7	Knockout	MZ-54 GBM cells	[57]
ATG5	knockout	TGS01 or TGS04	[55]
C14-IP-3	CRISPR-induced activation	LN229	[35]

## 6. Use of CRISPR-Cas9 Editing for Identifying Genetic Regulators of Angiogenesis in GBM

GBM is a highly vascular tumor characterized by new blood vessel formation or angiogenesis, which contributes to tumor rapid growth, invasiveness, and therapy resistance [66,67]. In response to signals from tumor cells or the surrounding microenvironment, new blood vessels are formed by proliferation and differentiation of blood vessel forming cells from different sources [67], for example, pre-existing endothelial cells, migratory endothelial or hemopoietic precursor cells, and GSCs [68,69]. CRISPR-Cas9 has recently been used to knockdown genes, which have been shown to regulate angiogenesis and it also has been used to identify angiogenesis-related novel prognostic biomarkers (Table 4).

An investigation used CRISPR-Cas9 to knockdown DDX39B (DExD-box helicase 39B) gene in U87MG cell line and found that down regulation of the expression of angiogenesis-related factors [70]. Similarly, it has been shown that CRISPR-Cas9 mediated knockdown of Notch1 gene significantly impaired expression of angiogenesis and related factors in response to radiotherapy in U87MG and U251 cells [33]. Besides, another group performed CRISPR-Cas9 mediated knockout of PDPN gene in several GBM cell types including GBMF2, GBMF3, LN308, and LN319. Although they found no changes in the rate of angiogenesis in knockouts compared with intact controls, expression of PDPN could be used as a tumor biomarker for unfavorable outcome [60].

## 7. CRISPR-Cas9 Editing of the Genes for Down Regulation of Cell Invasion and Migration in GBM

GBM cells can acquire resistant mesenchymal-like properties with activation of different pathways that promote local aggressive invasion and migration, which in turn contribute to incomplete surgical resection of the tumor, its recurrence, resistance to therapy, and lethality [71]. Also, GSCs are one of the major underlying causes of GBM invasion and resistance to treatment [9]. Overexpression and remodeling of extracellular matrix protein is another major cause of rapid dissemination of the disease and resistance to therapy [71,72,73]. CRISPR-Cas9 mediated gene editing in GBM cells has identified the critical molecules that impact invasion and migration positively or negatively (Table 5). A study used CRISPR-Cas9 mediated overexpression or knockdown of the oncoprotein doublecortin (DCX) in rat GBM C6 cells and found that knocking down of the DCX gene reduced invasion of GBM C6 cells [74]. Another study found that CRISPR-Cas9 mediated knockdown of the germline-related protein Dazl (deleted in azoospermia like) gene in the human GBM cell lines A172, U251, and LN229 decreased the abilities of cell invasion and migration [22].

Knockdown of the transcriptional regulator ATRX (alpha thalassemia/mental retardation syndrome X-linked) gene in GBM cells inhibited cell invasion [75]. In this study, it has been found that CRISPR-Cas9 mediated ATRX knockout leads to suppression of phosphorylation of ATM gene, which in turn inhibits the activation of the downstream regulatory proteins. Similarly, the knockdown of the tumor suppressor ATM (ataxia telangiectasia mutated) gene in the GBM LN18 cell line (p85α deficient) decreased cell invasion and migration, using corresponding in vitro assays [58]. Although this study notes that ATM and PI3K activations are important for cell invasion and migration, further studies are needed to explore the molecular and genetic mechanisms by which ATM regulates invasion and migration. Also, the investigators of this study found that ATM knockdown in LN229 cell line (p85α proficient) had no significant effect on cell invasion and migration. However, further studies are needed to understand how ATM interacts with p85α to regulate cell invasion and migration in different GBM cell lines. A study used CRISPR-Cas9 mediated knockout of the mesenchymal transcription factor ZEB1 (zinc finger E-box binding homeobox 1) gene, one of underlying causes of resistance to bevacizumab, attenuated invasion of GBM cells [76]. Another study found that Nanos3 (Nanos-family zinc finger protein 3) knockdown in GBM attenuated cell invasion and migration and enhanced responsiveness or sensitivity to doxorubicin (DOX) and temozolomide (TMZ) [41]. In contrast, a study found that knockdown of the transcription factor PAX6 (paired box protein 6) gene increased migration of human GBM U251 cells [77], while another study showed that podoplanin knockdown in GBM did not affect the invasiveness of these cells when compared with control cells [60]. Also, CRISPR-Cas9 was used to identify the role of cell surface receptors or related proteins in regulating GBM invasiveness. For example, knockdown of the cell surface receptors, neuropilin-1 (NRP1) and neuropilin-2 (NRP2), in U87MG cells showed their important roles in regulation of cytoskeleton contraction [42]. Knockdown of the aryl hydrocarbon receptor (AhR) gene in patient derived GBM cells enhanced expression of invasion and migration promoting genes [39]. Knockdown of caveolin-1 and cavin (caveolin-1 expression and cavin stability regulate caveolae dynamics) in GBM U251 cells decreased expression of matrix metalloproteinases (MMPs), epithelial mesenchymal markers, and epithelial–mesenchymal transition (EMT) markers and inhibited cell invasion [78].

In addition, CRISPR-Cas9 editing system has been used in studying the epigenetic regulators of GBM invasion and migration. A study has used CRISPR interference (CRISPRi) and CRISPR activation (CRISPRa) to show that the histone H3 Lys 27 demethylase KDM6B (Lys demethylase 6B) is involved in potentiating cell proliferation, invasion, and migration in U87MG and U251MG cells [79]. Another study showed that knockout of the TEA domain transcription factor1 (TEAD1) or TEAD4 reduced migration and EMT gene expression in GBM cells [80].

Furthermore, CRISPR-Cas9 aids in identifying genetic regulation of invasion and migration of GSCs. It was reported that deletion of RGS4 gene in GSCs decreased invasion and migration [64]. An investigation found that QKI (quaking homolog, KH domain RNA binding) gene knockout in GSCs improved invasiveness [81]. Knockdown of DDX39B in GSCs reduced expression of the factors related to extracellular matrix, cellular migration, and angiogenesis [70]. Knockdown of TP53 exon 4 in cerebral organoids derived from human embryonic stem cell (ESC) line rendered them more invasive [82].

**Table 5:** CRISPR-Cas 9 mediated knockouts of migration-related genes in GBM.

Target Gene	Type of CRISPR-Cas9 Mediated Genome Editing	GBM Model	References
QKI	Knockout	GSCs	[81]
PAX6	Knockout	U251	[77]
NRP1 and NRP2	Knockout	U87MG	[42]
ATRX	Knockdown	U251, LN229	[75]
TP53 exon 4	Homologous recombination to disrupt the TP53	Cerebral organoids of human embryonic stem cell line (H9)	[82]
C14-IP-3	CRISPR-induced activation	LN229	[35]
AhR	Knockdown	Patient-derived 15-037 cells	[39]
KDM6B	CRISPR interference (CRISPRi) and CRISPR activation (CRISPRa)	U87MG and U251	[79]
TEAD1 or TEAD4	Knockout	Patient-derived GBM cells	[80]
PDPN	Knockdown	GBMF3	[60]
DCX	Overexpression or knockdown	Rat C6 and human U251 cell lines	[74]
ZEB1	Knockdown	Bevacizumab-resistant xenograft models	[76]
Dazl	Knockdown	A172, U251, and LN229 cell lines	[22]
DDX39B	Knockdown	GBM34, GBM44, GSCs	[70]
RGS4	Knockout	GSC20 and GSC28	[64]
Nanos3	Knockdown	GBM	[41]
Caveolin-1 and cavin	Knockout	U251	[78]
ATM	Knockout	LN18 LN229	[58]

## 8. CRISPR-Cas9 Editing of the Inflammatory and Immune Response Genes in GBM

Aberrant inflammation and immune responses are correlated with high-grade astrocytoma and poor outcome [14,15]. Escaping immune response can result in failure to eradicate the genetically abnormal GBM cells and their progression to more aggressive phenotype [34]. Infiltration of macrophages in and around the tumor and their phenotypic polarization (M1 or M2) can influence tumor growth. Aberrant immune response in GBM can result from activation of immune inhibitory factors such as transforming growth factor beta (TGFβ) and prostaglandin, induction of immune cytotoxic signals, and promotion of macrophage phenotypic switch from pro-inflammatory M1 to anti-inflammatory M2 [14,15]. CRISPR-Cas9 mediated gene knockdown in GBM cell line aids in identifying critical molecules that contribute to abnormal immune response in GBM (Table 6). For example, CRISPR-Cas9 mediated osteopontin (OPN) gene deletion in GSCs increases cell sensitivity and cytotoxicity to CD8+ T cells and reduces attraction to M2 macrophages [40]. Also, CRISPR-Cas9 mediated knockdown of AIM2 (absent in melanoma 2), an inflammation related gene in GBM cells, reduces cell growth and increases sensitivity to TMZ [83]. In addition, another group showed that DDX39B knockout inhibited NF-κB pathway in U87MG cells [70].

## 9. CRISPR-Cas9 Editing of the Genes That Provide Proliferative Signals in GBM

Overexpression of growth promoting molecules or receptors can contribute to one of the major therapy resistance mechanisms in various cancers including GBM [11,84]. Overexpression of EGFR, activation of mTOR, and oncogenic miRNAs are some examples of aberrant proliferative signals that enhance GBM growth and resistance to therapy [11]. CRISPR-Cas9 editing for overexpression, knockout, and knockdown of genes have been used to discover and understand the function of the genes responsible for maintaining proliferative signals in GBM (Table 7). CRISPR-Cas9 mediated knockout and knockdown reveal the role of individual genes or their promotors, non-coding DNA, and/or translation regulators in sustaining the proliferation in GBM and their effects on GBM chemoresistance and radio-resistance. A group of investigators found that deletion of ID1 (inhibitor of DNA binding 1, a helix-loop-helix protein) gene in GBM cells reduced tumor growth and improved sensitivity to TMZ [85]. Another group used CRISPR-Cas9 mediated base editing of the mutated telomerase reverse transcriptase (TERT) promoter in GBM cells, and the results showed that it led to the reduced cell growth [86]. Deletion of AIM2 sensor protein in GBM cells increased cell proliferation and resistance to TMZ treatment [83]. Another study reported that Nanos3 knockdown using CRISPR-Cas9 in GBM cells reduced cell proliferation and increased cell sensitivity to DOX and TMZ [41]. A different study showed that knockdown of long non-coding DNA HOTAIRM1 (HOX antisense intergenic RNA myeloid 1) in GBM U251 cells reduced cell proliferation [87].

A group used CRISPR-Cas9 for overexpression or knockdown of DCX (doublecortin), a protein associated with intracellular microtubules, in GBM cells showing that overexpression of DCX potentiated GBM proliferation while knockdown of it reduced cell proliferation significantly [74]. Another study used CRISPR-Cas9 editing to delete a gene responsible for production of an integral constituent of centrioles, called pericentriolar material 1 (PCM1), from GBM cell lines. This study found that PCM1 played an important role in proliferation and chemoresistance of both GBM cells and GSCs, while its depletion enhanced the sensitivity of those cells to TMZ [88]. An investigation employed CRISPR-Cas9 editing to delete a gene called the RGS4, which acted as a negative regulator of G-protein signaling, in GSCs and found that its deletion inhibited cell growth [64]. Down regulation of tRNAiMet impedes proliferation and growth of GBM cells [89]. Knockdown of the transcription factor NRF2 (nuclear factor erythroid 2 related factor 2) in a model of U87MG neutrospheres reduces cell proliferation following radiation [90]. Dazl (deleted in azoospermia-like) knockdown in the GBM cell lines A172, U251, and LN229 reduces cell proliferation [22]. PAX6 knockout in U251 cell line enhances proliferation [77]. Liver X receptor beta (LXRβ) deletion in GBM cells arrests cell cycle and decreases cell survival [91]. Knockdown of the cyclin-dependent protein kinase 7 (CDK7) in U87MG and U251 cell lines impairs their proliferation [92]. In addition, knockdown of the enhancer between Ki67 (a marker of cell proliferation) and O6-methylguanine-DNA methyltransferase (MGMT) genes in SKMG3 cells impairs cell proliferation and enhances their sensitivity to TMZ treatment [23]. CRISPR-Cas9 mediated knockdown of the transcription factor STAT3 (signal transducer and activator of transcription 3) gene in GBM cells has no significant impact on cell proliferation in vitro; however, it has marked effect on inhibiting tumor growth in vivo [93].

CRISPR-Cas9 genome editing is also used to identify how cell membrane associated proteins or receptors impacts GBM growth. For example, CRISPR-Cas9 mediated knockout of Laminin-411 α4 and β1 chains in GBM cells reduces tumor growth in mice [94]. Laminin-411 is an important regulator of extracellular matrix and highly expressed in the tumor microenvironment of GBM and it has been found to regulate GBM growth in mice by signaling through Laminin-411–Notch pathway [94]. Proliferation of ciliated GBM cells were impaired by growing them in culture media obtained from CRISPR-Cas9 edited GBM cells to lack the transporter proteins KIF3A (kinesin family member 3A) or IFT88 (intraflagellar transport protein 88) [95]. Estrogen receptor beta (ERβ) knockout in U87MG regulated cell proliferation and growth [96]. Knockdown of the transmembrane chemokine receptor CXCR7 and the chemokine ligands CXCL16 and CX3CL1 in GBM cell line LN229 impacted cell growth and played a role in regulation of cellular dormancy in response to TMZ treatment [97].

CRISPR-Cas9 editing also discovered the role of epigenetic regulator in cell growth. CRISPRi or CRISPRa of KDM6B in GBM cells shows its important role in promoting proliferation of tumor cells [79]. Deletion of H3K27M (histone H3 Lys 27 mutant) from high-grade astrocytoma cell lines impaired proliferation and tumorigenesis [98]. SRSF3 (serine/arginine-rich splicing factor 3) knockout in GSCs reduces cell proliferation and survival [99]. In contrast, knockdown of podoplanin (PDPN) transmembrane protein does not affect tumor growth [60].

**Table 7:** CRISPR-Cas9 mediated knockouts of the proliferation-related genes in GBM.

Target Gene	Type of CRISPR-Cas9 Mediated Genome Editing	GBM Model	References
Laminin-411	Knockout	U87MG and LN229 and patient derived GBM cell lines TS543 and TS576	[94]
KIF3A and IFT88	Knockout	L0	[95]
KDM6B	CRISPR interference (CRISPRi) and CRISPR activation (CRISPRa)	U87MG and U251MG	[79]
AIM2	Knockout	U251MG	[83]
H3K27M	Knockout	HGG lines	[98]
PDPN	Knockout	GBMF2, GBMF3, and human GBM cell lines LN308 and LN319	[60]
SRSF3	Knockout	GSCs	[99]
ATM	Knockout	LN18 and LN229	[58]
DCX	High DCX expression or knockdown	Rat C6 and human U251MG	[74]
TERT promoter	Correction of mutated TERT promoter	U87, U251	[86]
tRNAiMet	Knockdown	U251	[89]
HOTAIRM1	Knockdown	U251MG	[87]
CXCR7 CXCL16 or CX3CL1	Knockouts	LN229	[97]
RGS4	Knockouts	GSC20 and GSC28	[64]
Nanos3	knockdown	A172, U251, and LN229	[41]
NRF2	Knockdown	U87MG neurospheres	[90]
Dazl	Knockdown	A172, U251, and LN229	[22]
CDK7	knockout	U87MG and U251MG	[92]
PCM1	Deletion	L0 and SN186 GBM cell lines	[88]
ID1	Deletion	GBM cell line	[85]
Enhancer between Ki67 and MGMT genes	Deletion of enhancer between Ki67 and MGMT genes	SKMG3	[23]
LXRβ	Deletion	GBM cell line	[91]
PAX6	Knockout	U251MG	[77]
STAT3	Knockout	MT330 GBM	[93]
ERβ	knockout	U87MG	[96]

## 10. CRISPR-Cas9 Editing of the Genes That Regulate Self-Renewal Capacity in GBM

GSCs belong to a subpopulation of the tumor cells that possess self-renewal capacity and responsible for GBM development, sustaining growth, therapy resistance, and recurrence [9,10]. When these subpopulation tumor cells are reactivated and self-renewed, they create more aggressive disease that resist conventional therapy [9,10]. These cells are genetically and phenotypically different from primary tumor and they resist radiotherapy, chemotherapy, or both by activating genetic and molecular mechanisms that help them resist cell death, improve their DNA repair activities, potentiate cell cycle and growth, or arrest growth at any stage and resume later [9,10]. Reports from our laboratory indicated that use of synergistic combination therapies could be an important avenue to attenuate self-renewal capacity and induce apoptosis in human GSCs [100]. Nowadays, CRISPR-Cas9 editing have been widely used for genome-wide screen, knock in, knockout, and knockdown of stem-cell related genes to understand their functions in GBM development and relapse. CRISPR-Cas 9 mediated genome-wide screens have been used to identify molecular signals maintaining GBM growth and stemness. Furthermore, CRISPR-Cas9 genome-wide screens with patient-derived GSCs and NSCs have been used to identify the set of genes that are normally present in neuronal precursors during development but reactivated in GBM [50].

CRISPR-Cas9 genome-wide screens using patient-derived GSCs revealed mutations that led to deregulation in redundant function of genes responsible for controlling mitotic activity in GBM, including PKMYT1 and WEE1 genes [50]. The results show that redundancy is lost between mitosis-regulatory genes (PKMYT1 and WEE1) in GSCs when compared with NSCs [50]. A group of investigators used CRISPR-Cas9 mediated whole-genome screening to identify molecular signals that maintain GSC growth and stemness and their interaction with macrophage [49]. CRISPR-Cas9 mediated knockouts and knockdowns have been also used to study the unique and redundant functions of stemness regulatory genes (Table 8). For example, CRISPR-Cas9 was used to knockout a gene called Dazl (deleted in azoospermia-like) in GBM cell lines [22]. Dazl is one of the germ cell genes that potentiate meiosis cell division and stemness, and CRISPR-Cas9 mediated Dazl deletion has resulted in downregulation of stem cell markers, reduction of cell growth, and increase in their sensitivity to DOX and TMZ [22].

A study used CRISPR-Cas9 knockout of FOXO3 (forkhead box O3) gene in U87MG cells, causing decreases in expression of the cancer stem cell markers Oct4 and Sox2 [101]. Another study has used CRISPR-Cas9 editing to delete FOXG1 (forkhead box G1) showing that deletion of FOXG1 in GBM cell line increases their differentiation to astrocytes [102]. Use of CRISPR-Cas9 editing to knockdown the transcription factor Nrf2 in U87MG neurospheres resulted in reduction of self-renewal and an increase in cell differentiation following radiation [90]. A group used CRISPR-Cas9 editing to knockout ALDH1A3 (aldehyde dehydrogenase 1 family member A3) gene in GSCs and GBM cell lines [103]. ALDH1A3 is an important aldehyde dehydrogenase responsible for metabolism of aldehydes to carboxylic acids [103]. ALDH1A3 knockdown resulted in increased sensitivity of the cells to TMZ treatment [103]. Also, use of CRISPR-Cas9 editing to knockout RGS4 (regulator of G-protein signaling 4) in GSCs resulted in reduction in growth of GSCs [64]. 

## 11. Conclusions, Limitations, and Future Directions

Besides its role in developing novel models for GBM as reviewed recently [104], CRISPR-Cas9 technology has offered novel insights into the unique and redundant roles of various genes in regulating proliferation, stemness, angiogenesis, and invasion of GBM cell lines, providing us promising therapeutic targets to treat or halt progression of this malignant disease. One of the major challenges of using this technology appeared obvious that viral Cas9 components were used in large number of GBM research, and this strategy could result in off-target editing and undesirable mutations. First, use of Cas9 nickase could provide more specific gene editing for limiting the off-target effect of the regular CRISPR-Cas9 that was widely used in most of these studies. Second, most of the CRISPR-Cas9 genome-wide screens in GBM research were performed using limited library and limited cell lines, which did not represent the whole spectra of the heterogenous nature of GBM. Third, more mechanistic studies are needed to explore the dual function of specific genes in pathogenesis of GBM and how it can regulate different hallmarks of this disease. Lastly, little is known about the in vivo effect of CRISPR-Cas9 mediated gene knockout or overexpression in GBM. Because majority of CRISPR-Cas9 genome editing were performed in GBM cell lines, whether primary in origin or derived from GSCs, more studies need to be conducted in animal models to evaluate and explore the effectiveness and reliability of this technology for translation of gene therapy to the clinics in targeting pathogenic hallmarks of GBM.

## Figures and Tables

**Table 4:** CRISPR-Cas9 mediated knockouts of angiogenesis genes in GBM.

Target Gene	Type of CRISPR-Cas9 Mediated Genome Editing	GBM Model	References
DDX39B	Knockdown	U87MG	[70]
PDPN	Knockout	GBMF2, GBMF3, LN308, LN319	[60]
Notch1	Knockdown	U87MG, U251	[33]

**Table 6:** CRISPR-Cas9 mediated knockouts of the inflammation genes in GBM.

Target Gene	Type of CRISPR-Cas9 Mediated Genome Editing	GBM Model	References
OPN	Knockout	GSCs	[40]
DDX39B	Knockout	U87MG	[70]
AIM2	Knockdown	U251	[83]

**Table 8:** CRISPR-Cas9 mediated knockouts of stem cells-related genes in GBM.

Target Gene	Type of CRISPR-Cas9 Mediated Genome Editing	GBM Model	References
FOXO3	Knockdown	U87MG	[101]
FOXG1	Deletion	GSCs	[102]
RGS4	Deletion	GSC20 and GSC28	[64]
ALDH1A3	Knockdown	LN229, U87MG, T98G, GSC-like cells T84 and X01	[103]
Dazl	Knockdown	A172, U251, and LN229	[22]
Nrf2	Knockdown	U87MG	[90]

## Data Availability

Not applicable.

## References

[1] De Eulate-Beramendi S.A., Piña-Batista K.M., Rodrigo V., Torres-Rivas H.E., Rial-Basalo J.C. (2016). Multicentric spinal cord and brain glioblastoma without previous craniotomy. Surg. Neurol. Int..

[2] Huse J.T., Holland E., De Angelis L.M. (2013). Glioblastoma: Molecular analysis and clinical implications. Annu. Rev. Med..

[3] Jacob G., Dinca E.B. (2009). Current data and strategy in glioblastoma multiforme. J. Med. Life.

[4] Johnson D.R., O’Neill B.P. (2012). Glioblastoma survival in the United States before and during the temozolomide era. J. Neurooncol..

[5] Inda M.M., Bonavia R., Seoane J. (2014). Glioblastoma multiforme: A look inside its heterogeneous nature. Cancers.

[6] van den Hengel S.K., Balvers R.K., Dautzenberg I.J., van den Wollenberg D.J., Kloezeman J.J., Lamfers M.L., Sillivis-Smit P.A., Hoeben R.C. (2013). Heterogeneous reovirus susceptibility in human glioblastoma stem-like cell cultures. Cancer Gene Ther. Sep..

[7] Haar C.P., Hebbar P., Wallace G.C., Das A., Vandergrift W.A., Smith J.A., Giglio P., Patel S.J., Ray S.K., Banik N.L. (2012). Drug resistance in glioblastoma: A mini review. Neurochem. Res..

[8] Ramirez Y.P., Weatherbee J.L., Wheelhouse R.T., Ross A.H. (2013). Glioblastoma multiforme therapy and mechanisms of resistance. Pharmaceuticals.

[9] Ortensi B., Setti M., Osti D., Pelicci G. (2013). Cancer stem cell contribution to glioblastoma invasiveness. Stem Cell Res. Ther..

[10] Auffinger B., Spencer D., Pytel P., Ahmed A.U., Lesniak M.S. (2015). The role of glioma stem cells in chemotherapy resistance and glioblastoma multiforme recurrence. Expert Rev. Neurother..

[11] Hatanpaa K.J., Burma S., Zhao D., Habib A.A. (2010). Epidermal growth factor receptor in glioma: Signal transduction, neuropathology, imaging, and radioresistance. Neoplasia.

[12] Valdés-Rives S.A., Casique-Aguirre D., Germán-Castelán L., Velasco-Velázquez M.A., González-Arenas A. (2017). Apoptotic signaling pathways in glioblastoma and therapeutic implications. Biomed. Res. Int..

[13] Taylor M.A., Das B.C., Ray S.K. (2018). Targeting autophagy for combating chemoresistance and radioresistance in glioblastoma. Apoptosis.

[14] Razavi S.M., Lee K.E., Jin B.E., Aujla P.S., Gholamin S., Li G. (2016). Immune evasion strategies of glioblastoma. Front. Surg..

[15] George J., Banik N.L., Ray S.K. (2009). Combination of hTERT knockdown and IFN-gamma treatment inhibited angiogenesis and tumor progression in glioblastoma. Clin. Cancer Res..

[16] Schulte J.D., Aghi M.K., Taylor J.W. (2020). Anti-angiogenic therapies in the management of glioblastoma. Chin. Clin. Oncol..

[17] Heuser V.D., Kiviniemi A., Lehtinen L., Munthe S., Kristensen B.W., Posti J.P., Sipilä J., Vuorinen V., Carpén O., Gardberg M. (2020). Multiple formin proteins participate in glioblastoma migration. BMC Cancer.

[18] Holland E.C. (2000). Glioblastoma multiforme: The terminator. Proc. Natl. Acad. Sci. USA.

[19] Simon T., Jackson E., Giamas G. (2020). Breaking through the glioblastoma micro-environment via extracellular vesicles. Oncogene.

[20] Jinek M., Chylinski K., Fonfara I., Hauer M., Doudna J.A., Charpentier E. (2012). A programmable dual-RNA-guided DNA endonuclease in adaptive bacterial immunity. Science.

[21] Nakazawa T., Natsume A., Nishimura F., Morimoto T., Matsuda R., Nakamura M., Yamada S., Nakagawa I., Motoyama Y., Park Y.S. (2020). Effect of CRISPR/Cas9-mediated PD-1-disrupted primary human third-generation CAR-T cells targeting EGFRvIII on in vitro human glioblastoma cell growth. Cells.

[22] Zhang F., Liu R., Zhang H., Liu C., Lu Y. (2020). Suppressing Dazl modulates tumorigenicity and stemness in human glioblastoma cells. BMC Cancer.

[23] Chen X., Zhang M., Gan H., Wang H., Lee J.H., Fang D., Kitange G., He L.J., Hu Z., Parney I.F. (2018). A novel enhancer regulates MGMT expression and promotes temozolomide resistance in glioblastoma. Nat. Commun..

[24] Yin H., Xue W., Anderson D.G. (2019). CRISPR-Cas: A tool for cancer research and therapeutics. Nat. Rev. Clin. Oncol..

[25] Marraffini L.A., Sontheimer E.J. (2010). CRISPR interference: RNA-directed adaptive immunity in bacteria and archaea. Nat. Rev. Genet. Mar..

[26] Horvath P., Romero D.A., Coûté-Monvoisin A.C., Richards M., Deveau H., Moineau S., Boyaval P., Fremaux C., Barrangou R. (2008). Diversity, activity, and evolution of CRISPR loci in *Streptococcus thermophilus*. J. Bacteriol..

[27] Hsu P.D., Lander E.S., Zhang F. (2014). Development and applications of CRISPR-Cas9 for genome engineering. Cell.

[28] Godde J.S., Bickerton A. (2006). The repetitive DNA elements called CRISPRs and their associated genes: Evidence of horizontal transfer among prokaryotes. J. Mol. Evol..

[29] Cong L., Ran F.A., Cox D., Lin S., Barretto R., Habib N., Hsu P.D., Wu X., Jiang W., Marraffini L.A. (2013). Multiplex genome engineering using CRISPR/Cas systems. Science.

[30] Mali P., Yang L., Esvelt K.M., Aach J., Guell M., DiCarlo J.E., Norville J.E., Church G.M. (2013). RNA-guided human genome engineering via Cas9. Science.

[31] Sander J.D., Joung J.K. (2014). CRISPR-Cas systems for editing, regulating, and targeting genomes. Nat. Biotechnol..

[32] Nishimasu H., Ran F.A., Hsu P.D., Konermann S., Shehata S.I., Dohmae N., Ishitani R., Zhang F., Nureki O. (2014). Crystal structure of Cas9 in complex with guide RNA and target DNA. Cell.

[33] Han N., Hu G., Shi L., Long G., Yang L., Xi Q., Guo Q., Wang J., Dong Z., Zhang M. (2017). Notch1 ablation radiosensitizes glioblastoma cells. Oncotarget.

[34] Yelton C.J., Ray S.K. (2018). Histone deacetylase enzymes and selective histone deacetylase inhibitors for antitumor effects and enhancement of antitumor immunity in glioblastoma. Neuroimmunol. Neuroinflamm..

[35] Nayak S., Aich M., Kumar A., Sengupta S., Bajad P., Dhapola P., Paul D., Narta K., Purkrait S., Mehani B. (2018). Novel internal regulators and candidate miRNAs within miR-379/miR-656 miRNA cluster can alter cellular phenotype of human glioblastoma. Sci. Rep..

[36] Hille F., Charpentier E. (2016). CRISPR-Cas: Biology, mechanisms and relevance. Philos. Trans. R. Soc. Lond. B Biol. Sci..

[37] Jiang W., Bikard D., Cox D., Zhang F., Marraffini L.A. (2013). RNA-guided editing of bacterial genomes using CRISPR-Cas systems. Nat. Biotechnol..

[38] Dudás A., Chovanec M. (2004). DNA double-strand break repair by homologous recombination. Mutat. Res..

[39] Jin U.H., Karki K., Cheng Y., Michelhaugh S.K., Mittal S., Safe S. (2019). The aryl hydrocarbon receptor is a tumor suppressor-like gene in glioblastoma. J. Biol. Chem..

[40] Wei J., Marisetty A., Schrand B., Gabrusiewicz K., Hashimoto Y., Ott M., Grami Z., Kong L.Y., Ling X., Caruso H. (2019). Osteopontin mediates glioblastoma-associated macrophage infiltration and is a potential therapeutic target. J. Clin. Investig..

[41] Zhang F., Liu R., Liu C., Zhang H., Lu Y. (2020). Nanos3, a cancer-germline gene, promotes cell proliferation, migration, chemoresistance, and invasion of human glioblastoma. Cancer Cell Int..

[42] Smolkin T., Nir-Zvi I., Duvshani N., Mumblat Y., Kessler O., Neufeld G. (2018). Complexes of plexin-A4 and plexin-D1 convey semaphorin-3C signals to induce cytoskeletal collapse in the absence of neuropilins. J. Cell Sci..

[43] Awah C.U., Chen L., Bansal M., Mahajan A., Winter J., Lad M., Warnke L., Gonzalez-Buendia E., Park C., Zhang D. (2020). Ribosomal protein S11 influences glioma response to TOP2 poisons. Oncogene.

[44] Huang K., Liu X., Li Y., Wang Q., Zhou J., Wang Y., Dong F., Yang C., Sun Z., Fang C. (2019). Genome-wide CRISPR-Cas9 screening identifies NF-κB/E2F6 responsible for EGFRvIII-associated temozolomide resistance in glioblastoma. Adv. Sci..

[45] Chow R.D., Guzman C.D., Wang G., Schmidt F., Youngblood M.W., Ye L., Errami Y., Dong M.B., Martinez M.A., Zhang S. (2017). AAV-mediated direct in vivo CRISPR screen identifies functional suppressors in glioblastoma. Nat. Neurosci..

[46] Prolo L.M., Li A., Owen S.F., Parker J.J., Foshay K., Nitta R.T., Morgens D.W., Bolin S., Wilson C.M., Vega L.J. (2019). Targeted genomic CRISPR-Cas9 screen identifies MAP4K4 as essential for glioblastoma invasion. Sci. Rep..

[47] Ye L., Park J.J., Dong M.B., Yang Q., Chow R.D., Peng L., Du Y., Guo J., Dai X., Wang G. (2019). In vivo CRISPR screening in CD8 T cells with AAV-Sleeping Beauty hybrid vectors identifies membrane targets for improving immunotherapy for glioblastoma. Nat. Biotechnol..

[48] MacLeod G., Bozek D.A., Rajakulendran N., Monteiro V., Ahmadi M., Steinhart Z., Kushida M.M., Yu H., Coutinho F.J., Cavalli F. (2019). Genome-wide CRISPR-Cas9 screens expose genetic vulnerabilities and mechanisms of temozolomide sensitivity in glioblastoma stem cells. Cell Rep..

[49] Tang M., Xie Q., Gimple R.C., Zhong Z., Tam T., Tian J., Kidwell R.L., Wu Q., Prager B.C., Qiu Z. (2020). Three-dimensional bioprinted glioblastoma microenvironments model cellular dependencies and immune interactions. Cell Res..

[50] Toledo C.M., Ding Y., Hoellerbauer P., Davis R.J., Basom R., Girard E.J., Lee E., Corrin P., Hart T., Bolouri H. (2015). Genome-wide CRISPR-Cas9 screens reveal loss of redundancy between PKMYT1 and WEE1 in glioblastoma stem-like cells. Cell Rep..

[51] Liu S.J., Malatesta M., Lien B.V., Saha P., Thombare S.S., Hong S.J., Pedraza L., Koontz M., Seo K., Horlbeck M.A. (2020). CRISPRi-based radiation modifier screen identifies long non-coding RNA therapeutic targets in glioma. Genome Biol..

[52] Morton A.R., Dogan-Artun N., Faber Z.J., MacLeod G., Bartels C.F., Piazza M.S., Allan K.C., Mack S.C., Wang X., Gimple R.C. (2019). Functional enhancers shape extrachromosomal oncogene amplifications. Cell.

[53] Hönscheid P., Datta K., Muders M.H. (2014). Autophagy: Detection, regulation and its role in cancer and therapy response. Int. J. Radiat. Biol..

[54] Chien C.H., Hsueh W.T., Chuang J.Y., Chang K.Y. (2019). Role of autophagy in therapeutic resistance of glioblastoma. J. Cancer Metastasis Treat..

[55] Vu H.T., Kobayashi M., Hegazy A.M., Tadokoro Y., Ueno M., Kasahara A., Takase Y., Nomura N., Peng H., Ito C. (2018). Autophagy inhibition synergizes with calcium mobilization to achieve efficient therapy of malignant gliomas. Cancer Sci..

[56] Fettweis G., Di Valentin E., L’homme L., Lassence C., Dequiedt F., Fillet M., Coupienne I., Piette J. (2017). RIP3 antagonizes a TSC2-mediated pro-survival pathway in glioblastoma cell death. Biochim. Biophys. Acta Mol. Cell Res..

[57] Zielke S., Meyer N., Mari M., Abou-El-Ardat K., Reggiori F., van Wijk S., Kögel D., Fulda S. (2018). Loperamide, pimozide, and STF-62247 trigger autophagy-dependent cell death in glioblastoma cells. Cell Death Dis..

[58] Ali R., Alabdullah M., Miligy I., Normatova M., Babaei-Jadidi R., Nateri A.S., Rakha E.A., Madhusudan S. (2019). ATM regulated PTEN degradation is XIAP E3 ubiquitin ligase mediated in p85α Deficient cancer cells and influence platinum sensitivity. Cells.

[59] Kranz D., Boutros M. (2014). A synthetic lethal screen identifies FAT1 as an antagonist of caspase-8 in extrinsic apoptosis. EMBO J..

[60] Eisemann T., Costa B., Harter P.N., Wick W., Mittelbronn M., Angel P., Peterziel H. (2019). Podoplanin expression is a prognostic biomarker but may be dispensable for the malignancy of glioblastoma. Neuro Oncol..

[61] Ranjan A., Srivastava S.K. (2017). Penfluridol suppresses glioblastoma tumor growth by Akt-mediated inhibition of GLI1. Oncotarget.

[62] Zhang J., Zhang C., Cui J., Ou J., Han J., Qin Y., Zhi F., Wang R.F. (2017). TRIM45 functions as a tumor suppressor in the brain via its E3 ligase activity by stabilizing p53 through K63-linked ubiquitination. Cell Death Dis..

[63] Rodvold J.J., Xian S., Nussbacher J., Tsui B., Cameron Waller T., Searles S.C., Lew A., Jiang P., Babic I., Nomura N. (2020). IRE1α and IGF signaling predict resistance to an endoplasmic reticulum stress-inducing drug in glioblastoma cells. Sci. Rep..

[64] Guda M.R., Velpula K.K., Asuthkar S., Cain C.P., Tsung A.J. (2020). Targeting RGS4 ablates glioblastoma proliferation. Int. J. Mol. Sci..

[65] Peng H., Du B., Jiang H., Gao J. (2016). Over-expression of CHAF1A promotes cell proliferation and apoptosis resistance in glioblastoma cells via AKT/FOXO3a/Bim pathway. Biochem. Biophys. Res. Commun..

[66] George J., Banik N.L., Ray S.K. (2009). Combination of taxol and Bcl-2 siRNA induces apoptosis in human glioblastoma cells and inhibits invasion, angiogenesis and tumour growth. J. Cell Mol. Med..

[67] Das S., Marsden P.A. (2013). Angiogenesis in glioblastoma. N. Engl. J. Med..

[68] Wang R., Chadalavada K., Wilshire J., Kowalik U., Hovinga K.E., Geber A., Fligelman B., Leversha M., Brennan C., Tabar V. (2010). Glioblastoma stem-like cells give rise to tumour endothelium. Nature.

[69] Lathia J.D., Mack S.C., Mulkearns-Hubert E.E., Valentim C.L., Rich J.N. (2015). Cancer stem cells in glioblastoma. Genes Dev..

[70] Szymura S.J., Bernal G.M., Wu L., Zhang Z., Crawley C.D., Voce D.J., Campbell P.A., Ranoa D.E., Weichselbaum R.R., Yamini B. (2020). DDX39B interacts with the pattern recognition receptor pathway to inhibit NF-κB and sensitize to alkylating chemotherapy. BMC Biol..

[71] Cheng L., Wu Q., Guryanova O.A., Huang Z., Huang Q., Rich J.N., Bao S. (2011). Elevated invasive potential of glioblastoma stem cells. Biochem. Biophys. Res. Commun..

[72] Mikhailova V., Gulaia V., Tiasto V., Rybtsov S., Yatsunskaya M., Kagansky A. (2018). Towards an advanced cell based. AIMS Genet..

[73] Belousov A., Titov S., Shved N., Garbuz M., Malykin G., Gulaia V., Kagansky A., Kumeiko V. (2019). The extracellular matrix and biocompatible materials in glioblastoma treatment. Front. Bioeng. Biotechnol..

[74] Ayanlaja A.A., Ji G., Wang J., Gao Y., Cheng B., Kanwore K., Zhang L., Xiong Y., Kambey P.A., Gao D. (2020). Doublecortin undergo nucleocytoplasmic transport via the RanGTPase signaling to promote glioma progression. Cell Commun. Signal..

[75] Han B., Cai J., Gao W., Meng X., Gao F., Wu P., Duan C., Wang R., Dinislam M., Lin L. (2018). Loss of ATRX suppresses ATM dependent DNA damage repair by modulating H3K9me3 to enhance temozolomide sensitivity in glioma. Cancer Lett..

[76] Chandra A., Jahangiri A., Chen W., Nguyen A.T., Yagnik G., Pereira M.P., Jain S., Garcia J.H., Shah S.S., Wadhwa H. (2020). Clonal ZEB1-driven mesenchymal transition promotes targetable oncologic antiangiogenic therapy resistance. Cancer Res..

[77] Hegge B., Sjøttem E., Mikkola I. (2018). Generation of a PAX6 knockout glioblastoma cell line with changes in cell cycle distribution and sensitivity to oxidative stress. BMC Cancer..

[78] Pu W., Qiu J., Nassar Z.D., Shaw P.N., McMahon K.A., Ferguson C., Parton R.G., Riggins G.J., Harris J.M., Parat M.O. (2020). A role for caveola-forming proteins caveolin-1 and CAVIN1 in the pro-invasive response of glioblastoma to osmotic and hydrostatic pressure. J. Cell Mol. Med..

[79] Sui A., Xu Y., Yang J., Pan B., Wu J., Guo T., Shen Y., Guo X. (2019). The histone H3 Lys 27 demethylase KDM6B promotes migration and invasion of glioma cells partly by regulating the expression of SNAI1. Neurochem. Int..

[80] Tome-Garcia J., Erfani P., Nudelman G., Tsankov A.M., Katsyv I., Tejero R., Zhang B., Walsh M., Friedel R.H., Zaslavsky E. (2018). Analysis of chromatin accessibility uncovers TEAD1 as a regulator of migration in human glioblastoma. Nat. Commun..

[81] Han B., Wang R., Chen Y., Meng X., Wu P., Li Z., Duan C., Li Q., Li Y., Zhao S. (2019). QKI deficiency maintains glioma stem cell stemness by activating the SHH/GLI1 signaling pathway. Cell Oncol..

[82] Ogawa J., Pao G.M., Shokhirev M.N., Verma I.M. (2018). Glioblastoma model using human cerebral organoids. Cell Rep..

[83] Chen P.A., Shrivastava G., Balcom E.F., McKenzie B.A., Fernandes J., Branton W.G., Wheatley B.M., Petruk K., van Landeghem F., Power C. (2019). Absent in melanoma 2 regulates tumor cell proliferation in glioblastoma multiforme. J. Neurooncol..

[84] Mohammad R.M., Muqbil I., Lowe L., Yedjou C., Hsu H.Y., Lin L.T., Siegelin M.D., Fimognari C., Kumar N.B., Dou Q.P. (2015). Broad targeting of resistance to apoptosis in cancer. Semin. Cancer Biol..

[85] Sachdeva R., Wu M., Smiljanic S., Kaskun O., Ghannad-Zadeh K., Celebre A., Isaev K., Morrissy A.S., Guan J., Tong J. (2019). ID1 is critical for tumorigenesis and regulates chemoresistance in glioblastoma. Cancer Res..

[86] Li X., Qian X., Wang B., Xia Y., Zheng Y., Du L., Xu D., Xing D., DePinho R.A., Lu Z. (2020). Programmable base editing of mutated TERT promoter inhibits brain tumour growth. Nat. Cell Biol..

[87] Shi T., Guo D., Xu H., Su G., Chen J., Zhao Z., Shi J., Wedemeyer M., Attenello F., Zhang L. (2020). HOTAIRM1, an enhancer lncRNA, promotes glioma proliferation by regulating long-range chromatin interactions within HOXA cluster genes. Mol. Biol. Rep..

[88] Hoang-Minh L.B., Deleyrolle L.P., Nakamura N.S., Parker A.K., Martuscello R.T., Reynolds B.A., Sarkisian M.R. (2016). PCM1 depletion inhibits glioblastoma cell ciliogenesis and increases cell death and sensitivity to temozolomide. Transl. Oncol..

[89] Yang J., Smith D.K., Ni H., Wu K., Huang D., Pan S., Sathe A.A., Tang Y., Liu M.L., Xing C. (2020). SOX4-mediated repression of specific tRNAs inhibits proliferation of human glioblastoma cells. Proc. Natl. Acad. Sci. USA.

[90] Godoy P.R.D.V., Pour Khavari A., Rizzo M., Sakamoto-Hojo E.T., Haghdoost S. (2020). Targeting NRF2, regulator of antioxidant system, to sensitize glioblastoma neurosphere cells to radiation-induced oxidative stress. Oxidative Med. Cell Longev..

[91] Patel D., Ahmad F., Kambach D.M., Sun Q., Halim A.S., Kramp T., Camphausen K.A., Stommel J.M. (2019). LXRβ controls glioblastoma cell growth, lipid balance, and immune modulation independently of ABCA1. Sci. Rep..

[92] Meng W., Wang J., Wang B., Liu F., Li M., Zhao Y., Zhang C., Li Q., Chen J., Zhang L. (2018). CDK7 inhibition is a novel therapeutic strategy against GBM both in vitro and in vivo. Cancer Manag. Res..

[93] Ganguly D., Fan M., Yang C.H., Zbytek B., Finkelstein D., Roussel M.F., Pfeffer L.M. (2018). The critical role that STAT3 plays in glioma-initiating cells: STAT3 addiction in glioma. Oncotarget.

[94] Sun T., Patil R., Galstyan A., Klymyshyn D., Ding H., Chesnokova A., Cavenee W.K., Furnari F.B., Ljubimov V.A., Shatalova E.S. (2019). Blockade of a Laminin-411-Notch axis with CRISPR/Cas9 or a nanobioconjugate inhibits glioblastoma growth through tumor-microenvironment cross-talk. Cancer Res..

[95] Hoang-Minh L.B., Dutra-Clarke M., Breunig J.J., Sarkisian M.R. (2018). Glioma cell proliferation is enhanced in the presence of tumor-derived cilia vesicles. Cilia.

[96] Liu J., Sareddy G.R., Zhou M., Viswanadhapalli S., Li X., Lai Z., Tekmal R.R., Brenner A., Vadlamudi R.K. (2018). Differential effects of estrogen receptor β isoforms on glioblastoma progression. Cancer Res..

[97] Adamski V., Hattermann K., Kubelt C., Cohrs G., Lucius R., Synowitz M., Sebens S., Held-Feindt J. (2020). Entry and exit of chemotherapeutically-promoted cellular dormancy in glioblastoma cells is differentially affected by the chemokines CXCL12, CXCL16, and CX3CL1. Oncogene.

[98] Harutyunyan A.S., Krug B., Chen H., Papillon-Cavanagh S., Zeinieh M., De J.N., Deshmukh S., Chen C.C.L., Belle J., Mikael L.G. (2019). H3K27M induces defective chromatin spread of PRC2-mediated repressive H3K27me2/me3 and is essential for glioma tumorigenesis. Nat. Commun..

[99] Song X., Wan X., Huang T., Zeng C., Sastry N., Wu B., James C.D., Horbinski C., Nakano I., Zhang W. (2019). SRSF3-Regulated RNA Alternative splicing promotes glioblastoma tumorigenicity by affecting multiple cellular processes. Cancer Res..

[100] Hossain M., Banik N.L., Ray S.K. (2012). Synergistic anti-cancer mechanisms of curcumin and paclitaxel for growth inhibition of human brain tumor stem cells and LN18 and U138MG cells. Neurochem. Int..

[101] Martinez E., Vazquez N., Lopez A., Fanniel V., Sanchez L., Marks R., Hinojosa L., Cuello V., Cuevas M., Rodriguez A. (2020). The PI3K pathway impacts stem gene expression in a set of glioblastoma cell lines. J. Cancer Res. Clin. Oncol..

[102] Bulstrode H., Johnstone E., Marques-Torrejon M.A., Ferguson K.M., Bressan R.B., Blin C., Grant V., Gogolok S., Gangoso E., Gagrica S. (2017). Elevated FOXG1 and SOX2 in glioblastoma enforces neural stem cell identity through transcriptional control of cell cycle and epigenetic regulators. Genes Dev..

[103] Wu W., Wu Y., Mayer K., von Rosenstiel C., Schecker J., Baur S., Würstle S., Liesche-Starnecker F., Gempt J., Schlegel J. (2020). Lipid peroxidation plays an important role in chemotherapeutic effects of temozolomide and the development of therapy resistance in human glioblastoma. Transl. Oncol..

[104] Koga T., Chen C.C., Furnari F.B. (2020). Genome engineering evolves brain tumor modeling. Neurol. Med. Chir..

